# Effect on Improving CO_2_ Sensor Properties: Combination of HPTS and γ-Fe_2_O_3_@ZnO Bioactive Glass

**DOI:** 10.1021/acsomega.3c05361

**Published:** 2023-10-16

**Authors:** Sibel Oguzlar, Merve Zeyrek Ongun, Aylin M. Deliormanlı

**Affiliations:** † Center for Fabrication and Application of Electronic Materials, Dokuz Eylul University, Izmir 35390, Turkey; ‡ Izmir Vocational High School, Chemistry and Chemical Processing Technologies Department, Chemical Technology Program, Dokuz Eylul University, Izmir 35210, Turkey; § Department of Metallurgical and Materials Engineering, Manisa Celal Bayar University, Manisa 45040, Turkey

## Abstract

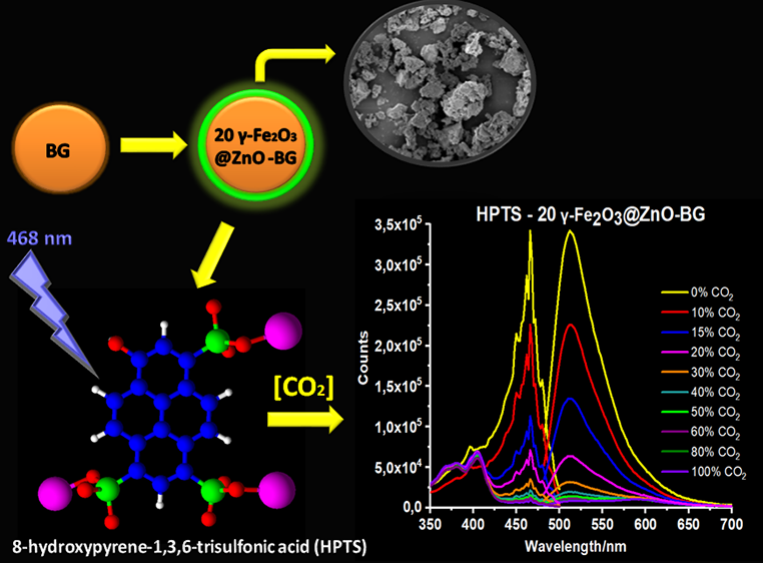

8-Hydroxypyrene-1,3,6-trisulfonic acid (HPTS) dye, a fluorescent dye often used as a pH indicator, is embedded within the bioactive glass matrix and undergoes changes in its fluorescent properties when exposed to carbon dioxide (CO_2_). The aim of the current study is to investigate the use of bioactive glass (BG) particles containing γ-Fe_2_O_3_@ZnO to enhance the CO_2_ sensitivity of HPTS. X-ray diffraction, Fourier transform infrared, scanning electron microscopy, and photoluminescence spectroscopies were used to characterize the sol–gel synthesized powders. The sensing slides were prepared in the form of a thin film by immobilizing the fluorescent dye and γ-Fe_2_O_3_@ZnO-based additives into the poly(methyl methacrylate) matrix. The addition of γ-Fe_2_O_3_@ZnO nanoparticles with bioactive glass additives to the HPTS improves the performance characteristics of the sensor, including the linear response range, relative signal variation, and sensitivity. Meanwhile, the CO_2_ sensitivities were measured as 10.22, 7.73, 16.56, 17.82, 19.58, and 42.40 for the undoped form and M, M@ZnO, 5M@ZnO-BG, 10M@ZnO-BG, and 20M@ZnO-BG NP-doped forms of the HPTS-based thin films, respectively. The response and recovery times of the HPTS-based sensing slide along with 20M@ZnO-BG NPs have been measured as 44 and 276 s, respectively. The γ-Fe_2_O_3_/ZnO-containing BG particle-doped HPTS composites can be used as a promising sensor agent in the detection of CO_2_ gas in various fields such as environmental monitoring, medical diagnostics, and industrial processes.

## Introduction

Monitoring the carbon dioxide (CO_2_) gaseous state is extremely useful in a variety of situations, including gas detection in the atmosphere, indoor environments, human breath, industrial plants, and automobile exhausts. Accurately and continuously measuring CO_2_ gas contribute to a better understanding and management of processes in biotechnology, chemical analysis, clinical analysis, and environmental monitoring.^1^ To determine the presence of CO_2_ gas, many methods based on infrared absorptiometry, electrochemistry, and luminescence can be utilized.^2^ Due to their benefits, such as electrical isolation, minimum noise interference, cheaper cost, quick reaction, adaptability for miniaturization, and ease of production and usage, optical sensors have become more common in recent years.^3^ There are several CO_2_-sensitive fluorescent dyes, which can be used for gas-sensing applications such as 8-hydroxypyrene-1,3,6-trisulfonic acid trisodium salt (HPTS),^4^ seminaphthorhodafluors (SNARFs),^5^ boron-dipyrromethene dyes (BODIPY),^6^ and metal–organic frameworks (MOFs),^7^ etc. The choice of the specific dye depends on factors such as the desired sensitivity, detection method, and compatibility with the sensing platform. HPTS, which exhibits significant emission and excitation bands when excited at 468 nm, is the most popular pH-sensitive dye.^4,8,9^

The use of polymeric matrices in optical sensors provides benefits such as encapsulation, protection, mechanical flexibility, permeability control, optical transparency, and ease of fabrication.^10^ Therefore, the fluorescent dye is physically trapped in polymeric matrices, such as silicone, ethyl cellulose (EC), polystyrene (PS), and poly(methyl methacrylate) (PMMA) in CO_2_ sensing agents. In this instance, the lifetime and intensity of the dye contained in the polymeric media both decrease due to the dye’s luminescent feature. Despite being often employed in optical applications and having a higher sensitivity to carbon dioxide, the HPTS has some limitations, such as stability, reproducibility, and low relative signal fluctuation. The HPTS dye has been coupled with a number of additives, such as ionic liquids (ILs), metal oxide semiconductors (MOSs), or coordination polymers (CPs), to get around these limitations.^11−13^

MOSs are widely used for gas-sensing applications due to their excellent performance in detecting various gases. Recent research has focused on doping metal oxide semiconductors with dissimilar metals or forming new composites to improve the performance and properties of metal oxide semiconductor gas sensors. Overall, n- or p-type metal oxide semiconductors offer a diverse range of gas-sensing functions due to their unique physical and chemical characteristics, compositions, and structures. Metal oxide semiconductors, such as ZnO (zinc oxide), SnO_2_ (tin dioxide), TiO_2_ (titanium dioxide), Co_3_O_4_ (cobalt oxide), Fe_2_O_3_ (iron oxide), and WO_3_ (tungsten trioxide), exhibit a wide range of gas-sensing functions. Recently, iron oxide nanostructures have gained significant attention in developing sensors due to their exceptional qualities that enhance sensor performance. Iron oxide, commonly known as hematite (α-Fe_2_O_3_), magnetite (Fe_3_O_4_), and/or maghemite (γ-Fe_2_O_3_), offers several benefits, including chemical and biological stability, low toxicity, a superparamagnetic characteristic, and low cost for large-scale synthesis that make it well suited for sensor applications.^14^ The choice of the shell material and its properties are critical considerations to achieve the desired performance and functionality of the Fe_2_O_3_ nanoparticles in biomedical imaging, drug delivery, magnetic separation, environmental remediation, and catalysis applications.^15^ Au,^16^ MoS_2_,^17^ MnO_2_,^18^ SiO_2_,^19^ ZnO,^20^ and TiO_2_^21^ have all been used for this purpose. Ahadpour Shal et al. reported that the coprecipitation technique was used to make core–shell-type magnetite Fe_3_O_4_@ZnO nanostructures.^22^ To improve the adherence of ZnO to the surface of Fe_3_O_4_, the surface was next modified using trisodium citrate. Hong et al. investigated the antioxidant capabilities of the ZnO shell by directly precipitating Fe_3_O_4_@ZnO core–shell particles on a nanoscale scale with zinc acetate and ammonium carbonate.^23^ Similarly, Wan et al. investigated the one-pot sequential polyol method for producing spherical magnetite@zinc oxide nanoparticles.^24^ Huarac and colleagues created Fe_3_O_4_@ZnO nanoparticles using Hong et al.’s method and examined photochemical activities.^25^ Li and colleagues combined atomic layer deposition and hydrothermal sintering to create γ-Fe_2_O_3_@ZnO core–shell photocatalysts. The resulting 3D core–shell structures exhibited magnetic separability and were designed for photocatalytic applications.^26^ In the study of Behera et al., the authors prepared γ-Fe_2_O_3_/ZnO nanocomposites to investigate their catalytic activity in the reduction of *p*-nitrophenol.^27^ A similar two-step coprecipitation technique was used by Loan et al. to create maghemite@ZnO core/shell nanocomposites in the presence of sodium citrate. The study likely focused on the structural characterization of the nanocomposites, including the determination of particle size, phase identification, and crystal structure analysis.^28^

In this study, the objective was to enhance the CO_2_ sensitivity of HPTS by incorporating γ-Fe_2_O_3_@ZnO nanoparticles with bioactive glass additives. The addition of bioactive glass (BG) particles to the γ-Fe_2_O_3_@ZnO material has several effects on the structure, leading to improvements in certain properties. When added to the γ-Fe_2_O_3_/ZnO material, these BG particles can introduce additional porosity to the composite structure because of their porous structure. The increased porosity can enhance the surface area of the material, providing more active sites for chemical reactions, gas adsorption, or other surface-dependent processes. The cohesive interaction between the BG particles and the γ-Fe_2_O_3_/ZnO matrix can help prevent structural deformation or particle agglomeration, leading to improved material integrity and durability. The CO_2_ intensity-based emission spectra and decay kinetics of the HPTS-based composites, including γ-Fe_2_O_3_/ZnO-containing BG particles encapsulated in the PMMA matrix, were analyzed.

## Experimental Section

### Reagents

The chemicals used to synthesize maghemite (γ-Fe_2_O_3_), iron(III) chloride (FeCl_3_ × 6H_2_O), iron(II) chloride tetrahydrate (FeCl_2_ × 4H_2_O), iron(III) nitrate nonahydrate (Fe(NO_3_)_3_ × 9H_2_O), ammonium hydroxide solution, and nitric acid (HNO_3_), were from Sigma-Aldrich. Zn(CH_3_COO)_2_ × 2H_2_O and ethylene diamine tetraacetic acid (EDTA) were used from Sigma-Aldrich, without further purification. On the other hand, tetraethyl orthosilicate, triethyl phosphate, sodium nitrate, calcium nitrate tetrahydrate, magnesium nitrate hexahydrate, and potassium nitrate (all from Sigma-Aldrich) were used for the synthesis of silicate-based 13–93 bioactive glass (53SiO_2_, 6Na_2_O, 12K_2_O, 5MgO, 20CaO, 4P_2_O_5_, wt %) particles containing ZnO-coated maghemite core–shell nanostructures. All of the compounds were analytically pure and used without further purification.

The following materials were supplied from Sigma-Aldrich: tetrahydrofuran (THF) and ethanol (EtOH) (99.8% by volume) as solvents, dioctylphatalate (DOP) as a plasticizer, 1-butyl-3-methyl imidazolium tetrafluoroborate (BMIMBF_4_) as the ionic liquid, and poly(methyl methacrylate) (PMMA) as a polymeric membrane. N_2_ and CO_2_ gas cylinders from Tinsa Gas in Izmir, Turkey, had a purity of 99.9%. 8-Hydroxypyrene-1,3,6-trisulfonic acid trisodium salt (HPTS-CO_2_-sensitive and fluorescent dye), dichloromethane (CH_2_Cl_2_), and tetraoctyl ammonium bromide (TOABr) were from Sigma-Aldrich and used in the form of ion pairs synthesized in previous studies. In a 1% sodium carbonate solution and CH_2_Cl_2_ (1:1), the trisodium salts of HPTS and TOABr were combined in a 1:4 ratio. The production of the ion pair required the use of a separating funnel, and the organic solvent was evaporated to obtain the ion pair.^29^

### Instrumentation

A scanning electron microscope (Zeiss, GeminiSEM 560) and a transmission electron microscope (FEI Tecnai G2 Spirit BioTwin CTEM, 120 kV) were used to examine the morphology of the produced particles. Prior to scanning electron microscopy (SEM) studies, gold–palladium was sputter-coated on all samples. A particle size analyzer (Malvern, Master Sizer 3000, UK) was used to measure the particle size distribution of the as-prepared bioactive glass powders. A Fourier transform infrared (FTIR) spectrometer (Thermo Fisher Scientific, Nicolet IS20, USA) was used to examine the structural characteristics between 525 and 4000 cm^–1^. Using a diffractometer (X-ray diffraction, XRD, Malvern Panalytical, Empyrean), the produced powders’ crystalline structure was examined. Samples were examined using a Cu–K X-ray tube with a scanning speed of 0.01°/min in the two-theta range of 10–90°. A spectrofluorometer system with a red-sensitive photomultiplier tube (FLSP920 Fluorescent Spectrometer, Edinburgh Instruments) was used for steady-state photoluminescence (PL) measurements. Using the FLSP920s time-related single photon counting mode (TCSPC), the decay time values were measured. A Sonimix 7000A gas mixing device was used to combine CO_2_ and N_2_ gases in a concentration range of 0–100% for detection measures. Under the operating ambient circumstances, gas mixtures were introduced to the system by immersing a diffuser needle in the sensing medium.

### Synthesis of (M@ZnO) Core@Shell Nanoparticles

The synthesis of maghemite (γ-Fe_2_O_3_) was carried out using the coprecipitation method.^30^ For this purpose, with a molar ratio of 2:1, the salts of ferric chloride and ferrous chloride were dissolved in ultrapure water using a magnetic stirrer for 30 min. After it had completely dissolved, ammonium hydroxide solution (25 wt %) was added to the mixture. The solid phase of the system was separated using a Nd magnet after 20 min of magnetic stirring at around 1600 rpm. Then, the pH of the system was dropped from about 10.5 to 8.0 by washing with pure water. Subsequently, the liquid medium was fully removed following the washing of the system made up of Fe_3_O_4_ nanoparticles, and then 0.21 M Fe(NO_3_)_3_ and 0.5 M HNO_3_ were added. This mixture was stirred with a magnetic stirrer at 90 °C for 1 h. The system was cooled to room temperature and fractionated with a Nd magnet, and the liquid portion on the top surface of the nanoparticles was transferred. The nanoparticle system was added to a tubular membrane made of cellulose after it was mixed with ultrapure water. This system was dialyzed in 0.01 M HNO_3_ for 2 days and then centrifuged for 15 min at 9000 rpm. Centrifuging was used to separate the obtained nanoparticles, which were then washed three times in ethanol and ultrapure water before being dried for 18 h at 60 °C.

For the preparation of maghemite-zinc oxide (M@ZnO) core–shell nanostructures, Zn(CH_3_COO)_2_ × 2H_2_O and EDTA were utilized, and the details of the coating procedure have been described in detail elsewhere.^31^ The coating was made at a fixed γ-Fe_2_O_3_:Zn-acetate ratio (1:6). For this purpose, γ-Fe_2_O_3_ in ethanol and Zn-acetate + EDTA (1 mol) in ethanol were mixed separately for 15 h. After that, the solutions were combined, stirred for 30 min using a magnetic stirrer, and homogenized for 10 min using an ultrasonic probe. NaOH was gradually added to the mixture to continue the reaction until the pH was 8.20. A magnetic stirrer was used to mix the solution for 3 h. Then, stirring was carried out for an additional hour at 75 °C. For 10 min, the precipitate was centrifuged at 9000 rpm. It was then rinsed twice: once with ethanol and once with ultrapure water and dried for 24 h at 60 °C.

### Synthesis of Bioactive Glass Particles Containing M@ZnO

In the study, silicate-based 13–93 bioactive glass particles containing ZnO-coated maghemite core–shell nanostructures at different concentrations were prepared using the sol–gel method.^32^ To accomplish this goal, ultrapure water and HNO_3_ were placed in a glass bottle and mixed for 20 min. Following that, tetra ethyl ortho silicate, triethyl phosphate, sodium nitrate, calcium nitrate tetrahydrate, magnesium nitrate hexahydrate, and potassium nitrate were added in the order listed. Subsequently, M-ZnO nanopowders were incorporated into the glass solution at different concentrations, namely, 5, 10, and, 20 wt %, and stirred for 18 h with a magnetic stirrer before being homogenized for 15 min with an ultrasonic probe. The gelation process was carried out in an incubator set to 37 °C for 5 days. The gelled system was aged in an oven at 60 °C for 48 h before drying at 120 °C. The dried bioactive glass particles were calcined in an air atmosphere for 4 h at 625 °C (heating rate 5 °C/min). An agate mortar was utilized to grind the particles for size reduction, and then, they were sieved below 38 μm. Fabricated bioactive glass composite powders containing 5, 10, and 20 wt % M-ZnO nanoparticles were designated as 5M@ZnO-BG, 10M@ZnO-BG, and 20M@ZnO-BG, respectively.

Bioactive glass particles often possess a porous structure characterized by interconnected voids and channels. When added to γ-Fe_2_O_3_@ZnO material, these BG particles can introduce additional porosity to the composite structure. The increased porosity can enhance the surface area of the material, providing more active sites for chemical reactions, gas adsorption, or other surface-dependent processes. This increased surface area can improve the material’s performance in applications such as catalysis or sensing, where a high surface-to-volume ratio is desirable. Overall, the addition of bioactive glass particles to γ-Fe_2_O_3_@ZnO material can bring about structural improvements such as increased porosity, enhanced stability, controlled release properties, and surface functionalization.^33,34^

### Thin Film Preparation

The carbon dioxide (CO_2_) detection solid membranes that were employed in the investigation were made from the same materials. The preparation of the polymer-based sensing thin films involved combining 100 mg of PMMA as the polymer, 96 mg of DOP as the plasticizer, 24 mg of [BMIM][BF_4_] as the ionic liquid, 2.5 mL of THF as the solvent, and 0.10 mg of dye as the ion pair form. Additive materials (0.10 mg) were individually added to the polymer-based cocktails to increase the CO_2_ sensitivity of HPTS. The components for the HPTS-based cocktail, both with and without additions, are shown in Table 1. The generated composites were here placed on a polyester substrate (of the Mylar TM variety) using the knife coating method, and a Tencor Alpha Step 500 profilometer was utilized to measure the thickness of these thin films. The results of the average thickness measurement were 6.35 ± 0.12 mm (*n* = 5). The thin films were placed in a quartz cuvette for optical measurements.

**Table 1:** Cocktail Compositions of the CO_2_-Sensitive HPTS-Based Composites

sample	additive (mg)	PMMA (mg)	THF (mL)	dye-HPTS (mg)
HPTS		100	2.5	0.1
M	0.1	100	2.5	0.1
M@ZnO	0.1	100	2.5	0.1
5M@ZnO-BG	0.1	100	2.5	0.1
10M@ZnO-BG	0.1	100	2.5	0.1
20M@ZnO-BG	0.1	100	2.5	0.1

## Results and Discussion

### Morphological, Structural, and Elemental Characteristics of the Powders

**Figure 1:**
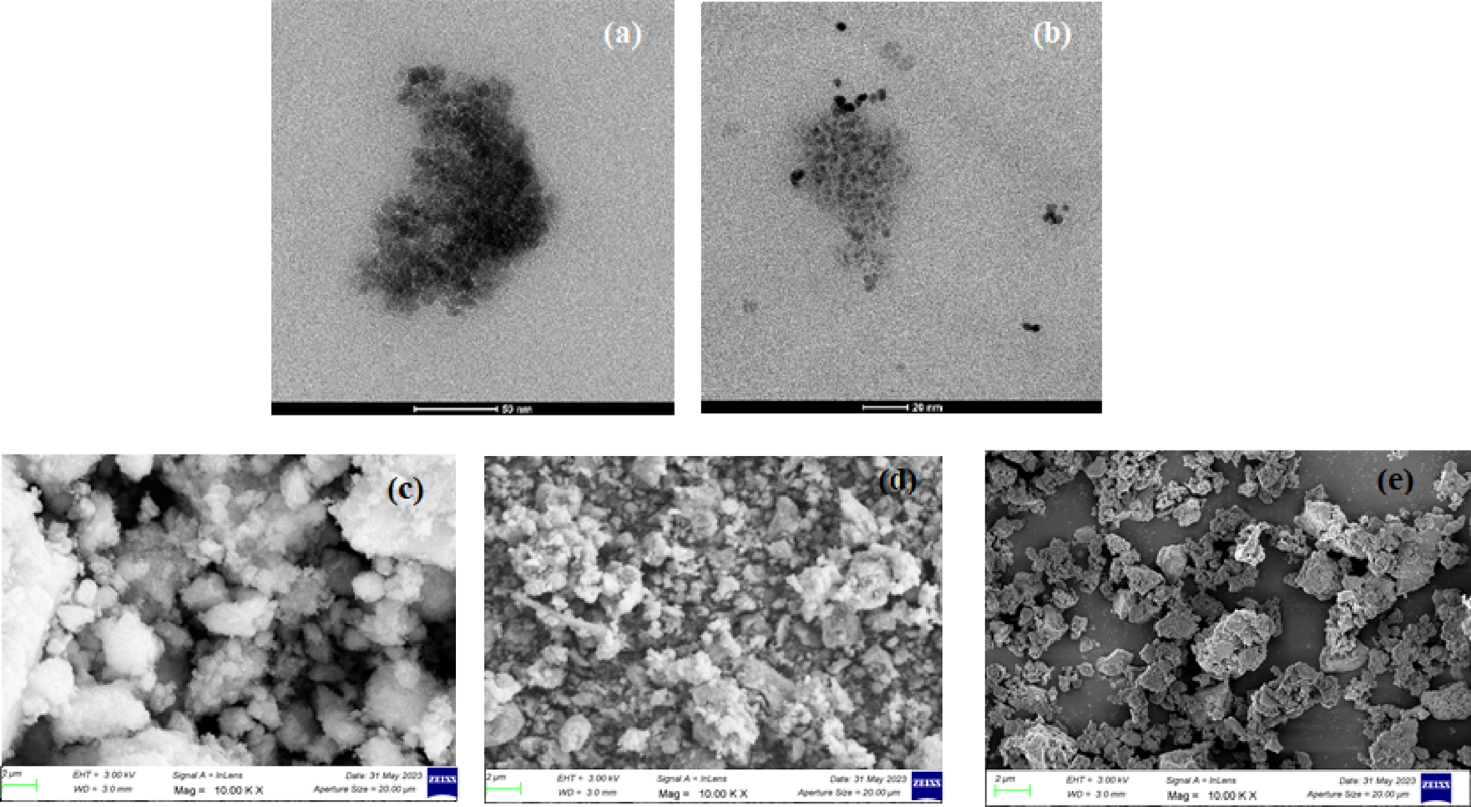
TEM images of the (a) M and (b) M@ZnO; SEM micrographs of the (c) M, (d) M@ZnO, and (e) 20M@ZnO-BG.

Transmission electron microscopy (TEM) analysis results of synthesized pristine γ-Fe_2_O_3_ and γ-Fe_2_O_3_@ZnO core–shell nanoparticles are given in Figure 1a–d. With the use of ImageJ application, TEM pictures were processed to determine the average particle size. The pristine γ-Fe_2_O_3_ powders and the γ-Fe_2_O_3_@ZnO nanostructures were found to have particle sizes of 6.75 and 8.14 nm, respectively.

**Figure 2:**
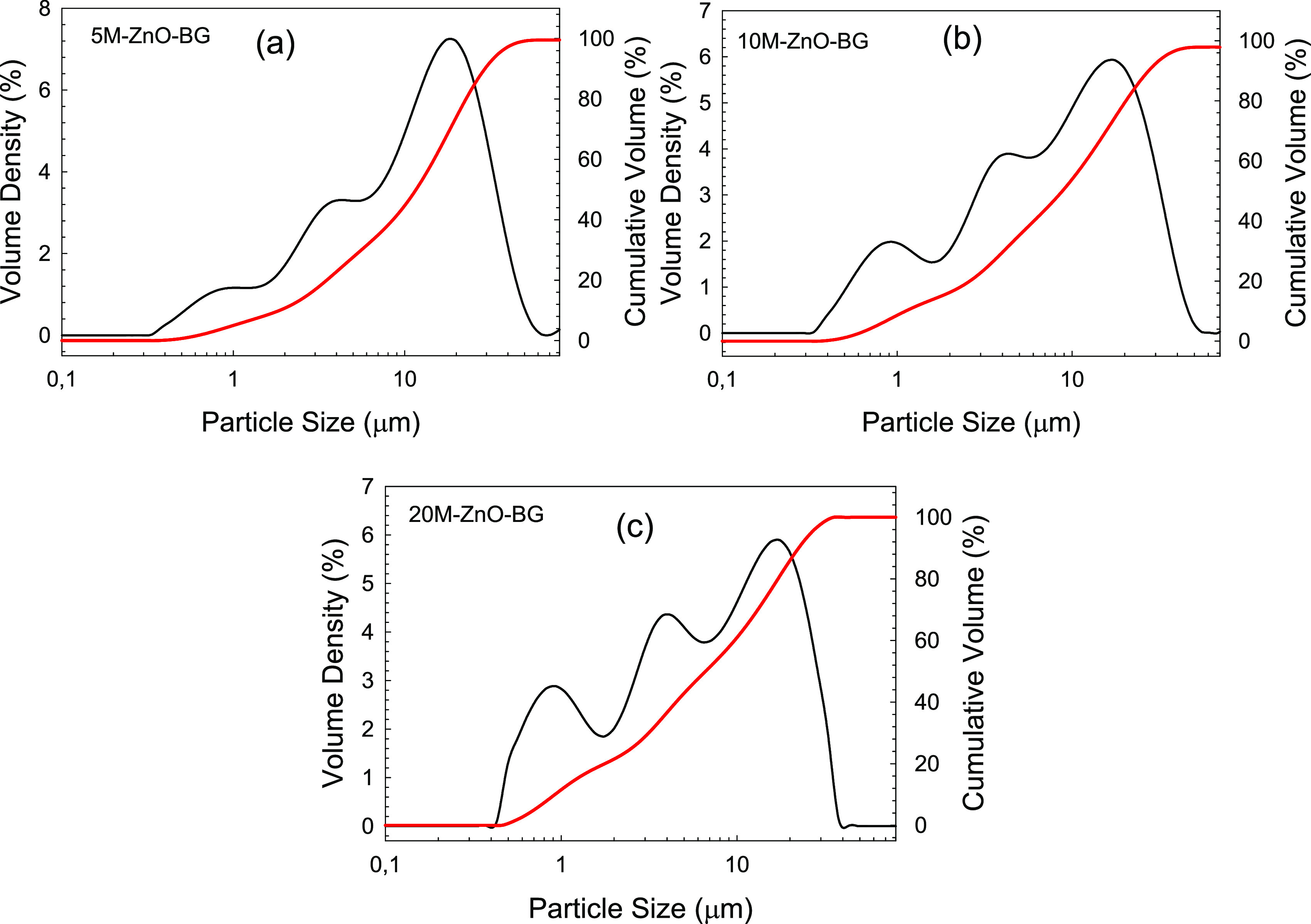
Particle size of the (a) 5M@ZnO-BG, (b) 10M@ZnO-BG, and (c) 20M@ZnO-BG powders. The red line represents cumulative volume (%).

TEM pictures of the samples also demonstrated that maghemite nanoparticles have a propensity to aggregate, whereas ZnO-coated powders displayed better dispersion behavior and were uniformly distributed. According to earlier publications, the critical diameter for the monodomain magnetic structure was 90 nm, while for the γ-Fe_2_O_3_ particles, superparamagnetic behavior can be seen below 30 nm.^35,36^ SEM micrographs of the fabricated particles including the γ-Fe_2_O_3_@ZnO-containing (20 wt %) bioactive glass powders are given in Figure 1c,d. SEM micrographs also indicated the better dispersion ability of the ZnO-coated maghemite nanoparticles compared with the uncoated powders. Both γ-Fe_2_O_3_ and γ-Fe_2_O_3_@ZnO core–shell nanoparticles demonstrated spherical morphology; on the other hand, bioactive glass particles containing γ-Fe_2_O_3_@ZnO have an irregular shape. Particle size distribution analysis of the prepared bioactive glasses also indicated the presence of a multimodal size distribution (Figure 2). A decrease in particle size of the bioactive glass powders was obtained as the concentration of the magnetic nanoparticles increased. Accordingly, the median particle sizes of the bioactive glass powders containing 5, 10, and 20 wt % magnetic nanoparticles were measured as 11.6, 8.80, and 6.63 μm, respectively (Table 2).

**Table 2:** Median Particle Size of the Bioactive Glass Powders

**sample**	***d***_**10**_**(μm)**	***d***_**50**_**(μm)**	***d***_**90**_**(μm)**
5M@ZnO-BG	1.86	11.6	29.5
10M@ZnO-BG	1.12	8.80	27.4
20M@ZnO-BG	0.92	6.63	22.5

**Figure 3:**
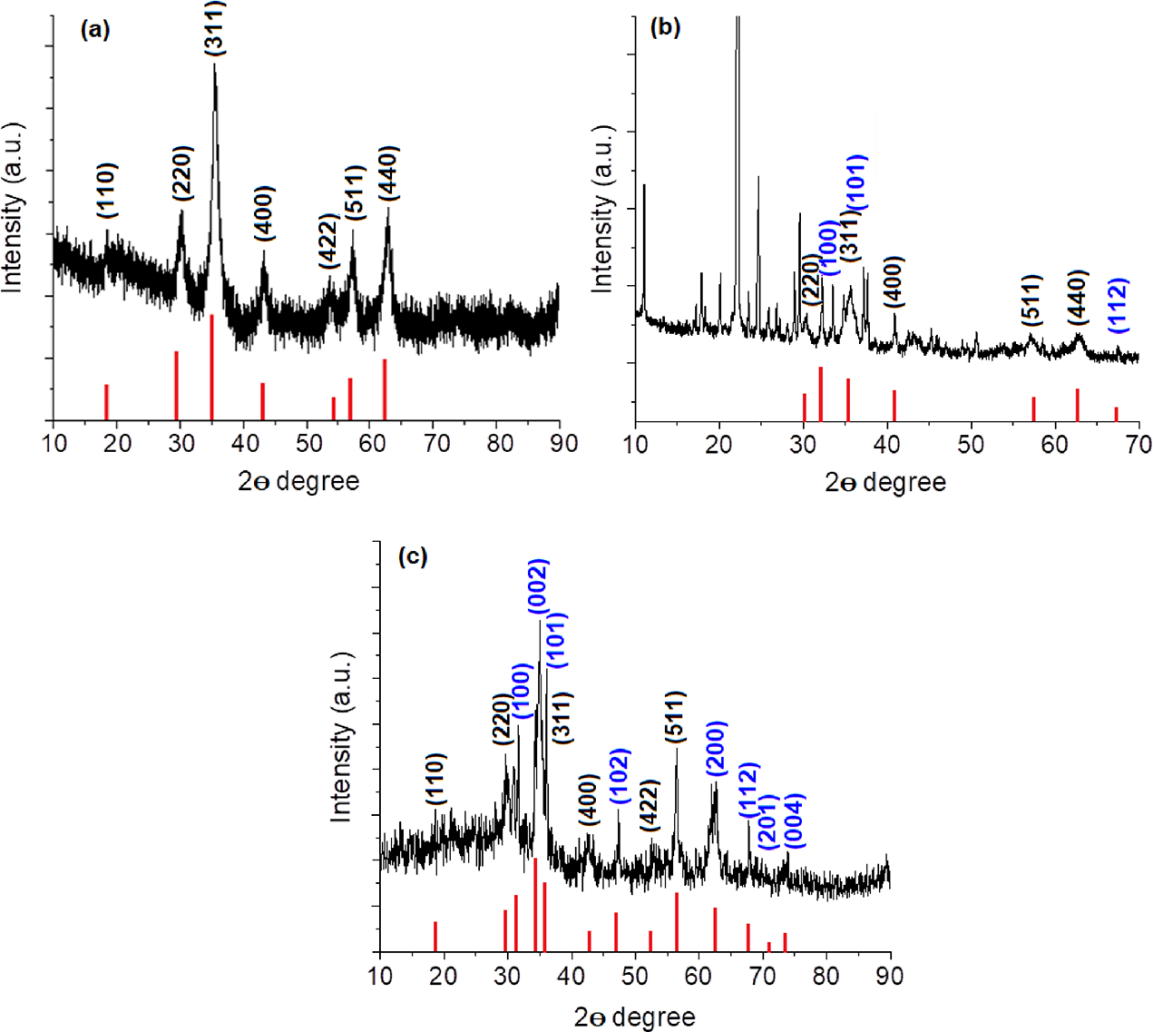
XRD patterns of the (a) M, (b) M@ZnO, and (c) 20M@ZnO particles.

XRD and FTIR analyses were used to examine the structural characteristics of the analyzed particles. The produced γ-Fe_2_O_3_ nanopowders’ XRD patterns are shown in Figure 3a. All of the distinctive peaks of maghemite are present in the XRD pattern of the produced Fe_2_O_3_ nanoparticles, and the pattern matches the peaks in JCPDS PDF file no. 00-039-1346.^37^ The crystalline planes of (220), (311), (400), (511), and (440) in the pattern are connected to the diffraction peaks at two-theta of 30.2, 35.5, 43.2, 57.3, and 62.8, respectively. The ZnO peaks, in addition to the peaks denoting the iron oxide core, can be seen in the XRD pattern of the ZnO-coated nanoparticles. As a result, the peaks at 31.7° (100), 34.2° (002), 35.6° (101), 48.1° (102), 62.5° (200), 67.9° (112), 69.0° (201), and 72.6° (004) were attributed to ZnO (JCPDS 36-1451).^38^ The ZnO peak (110) and γ-Fe_2_O_3_ peak (511), as well as the ZnO peak (103) and (440) maghemite peak, overlap at 57 and 62.5°, respectively. The presence of both maghemite and ZnO phases in the structure is shown by the XRD pattern of the magnetic nanoparticle-containing bioactive glass powders. Results further showed that the 13–93 glass structure did not further crystallize due to the presence of ZnO-coated maghemite nanoparticles.

**Figure 4:**
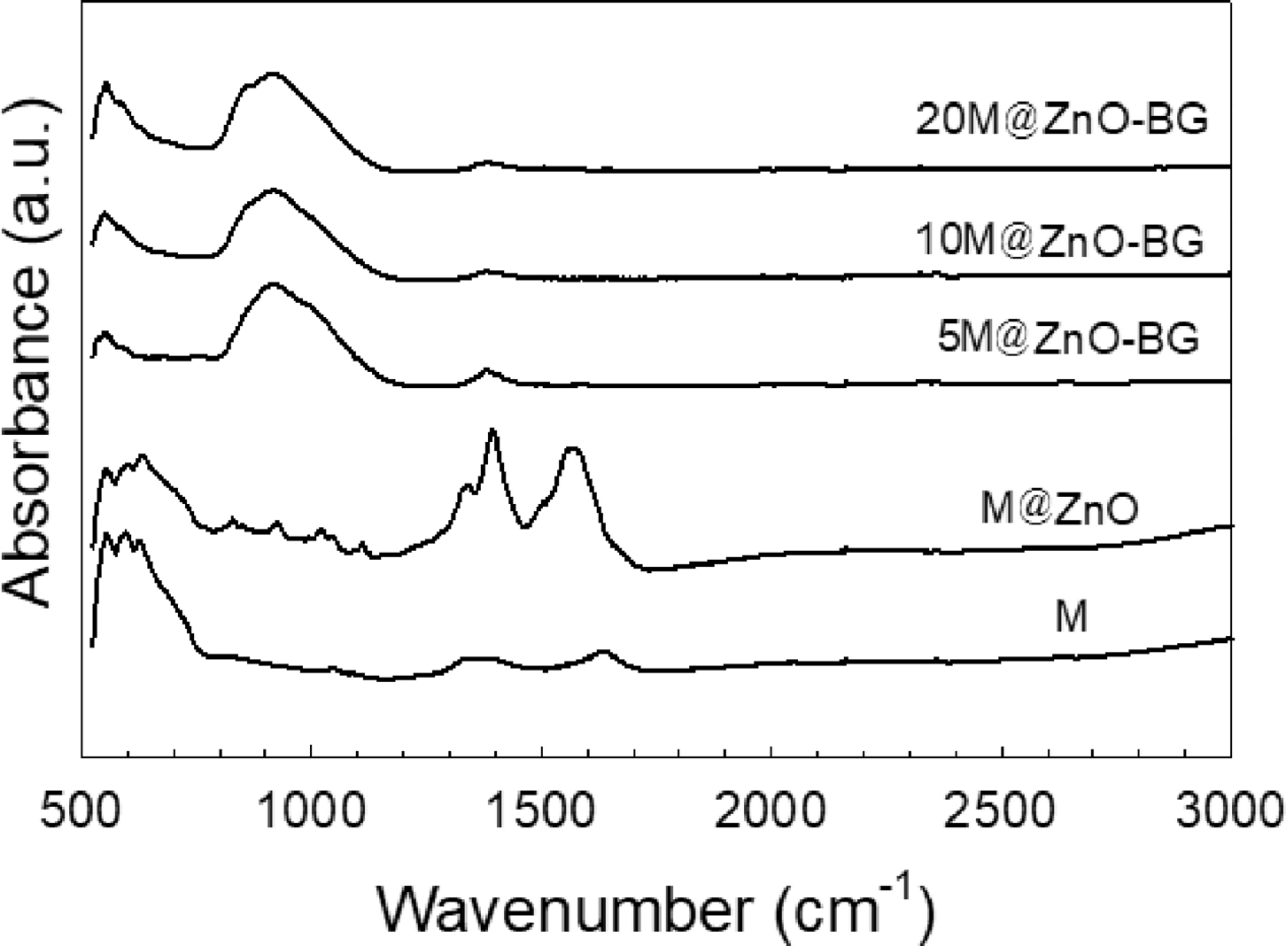
FTIR spectra of the particles synthesized in the study.

The FTIR spectra of the particles under investigation are shown in Figure 4. The FTIR spectrum of γ-Fe_2_O_3_ exhibits characteristic absorption bands at 630, 598, and 555 cm^–1^ that are attributed to iron–oxygen deformations (Fe–O) in the tetrahedral and octahedral regions.^39^ The stretching vibration band of O–N–O associated with the FeNO_3_ utilized as a precursor in the production of γ-Fe_2_O_3_ is also visible at 1384.36 cm^–1^. Additionally, the bending vibration bands carbon–hydrogen and carbon–carbon are represented by the absorption peaks at 2930 and 1075 cm^–1^, respectively. Zn–O bonds are represented by peaks at 520, 613, 674, and 1330 cm^–1^ in the FTIR spectra. The Zn–O–Zn bonds are responsible for the peak at 650 cm^–1^.^40^ The FTIR spectra of the bioactive glasses with magnetic nanoparticles revealed an asymmetric Si–O absorption peak at 929 cm^–1^. Additionally, it is seen that when the magnetic phase content of the bioactive glass matrix increases, so do the peaks corresponding to maghemite.

### CO_2_ Response of HPTS Thin Films Containing M@ZnO-Based Additives

The objective of this study is to examine how the inclusion of M@ZnO-based particles as additives in the PMMA matrix affects the CO_2_ sensitivity of HPTS. The process involves exposing the thin films to gas samples with varying concentrations of CO_2_, ranging from 0 to 100%. The CO_2_ gas is humidified, which allowed the formation of carbonic acid. This is important because carbonic acid must reach the active regions of HPTS for the measurement. In general, higher humidity levels will lead to a more significant formation of carbonic acid and, therefore, a more pronounced response from the sensor. HPTS is an indicator dye used for pH measurement and is sensitive to changes in the concentration of hydrogen ions (H^+^), which is influenced by the presence of carbonic acid. When carbonic acid forms due to the humidification of CO_2_ gas, it contributes to changes in the pH of the solution containing HPTS.

In the mechanism of the CO_2_ response of HPTS-based thin films along with additives, the anionic (deprotonated) form of the HPTS (Dye) is often stabilized in the PMMA matrix by the addition of the ionic quaternary ammonium base (TOA^+^OH^–^), a counterion. The sensing strategy in such designs is based on two processes: the first is the diffusion of CO_2_ into the detecting zone across the membrane, and the second is the reaction of the gas with an anionic, highly fluorescent HPTS phenolate anion. The indicator works because the ion pair’s water content results in the creation of carbonic acid when CO_2_ is present. The fluorescence of HPTS at 515 nm is decreased by the hydration of CO_2_ and subsequent protolysis, which also changes the fluorescent HPTS anion (Dye^–^) into less luminous HPTS (Dye). The reaction is described by the equation below^29^ (see eq 1):
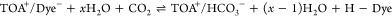
1

As the concentration of CO_2_ increases in the gas sample, the HPTS dye undergoes a change in its fluorescent properties. Initially, the dye emits a green fluorescence, and with the increase in the CO_2_ concentration, the dye transitions into a less fluorescent form, resulting in a light yellow color. Specifically, a drop in the emission signal is observed in the emission band at a wavelength of 515 nm. The Stern–Volmer equation is a fundamental equation used in fluorescence quenching studies to describe the relationship between the fluorescence intensity of a fluorophore and the concentration of a quenching species (eq 2). It assumes a dynamic quenching process, where the quenching species interacts with the excited state of the fluorophore, reducing its emission intensity. The equation is as follows:
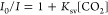
2where *I* stands for the fluorophore’s fluorescence intensity when the quenching species is present at the concentration [*Q*]; *I*_0_ represents the fluorophore’s fluorescence intensity when the quenching species is absent; *K*_sv_ is the Stern–Volmer quenching constant, which measures the effectiveness of the quenching procedure; and the quenching species’ concentration is [CO_2_].

**Figure 5:**
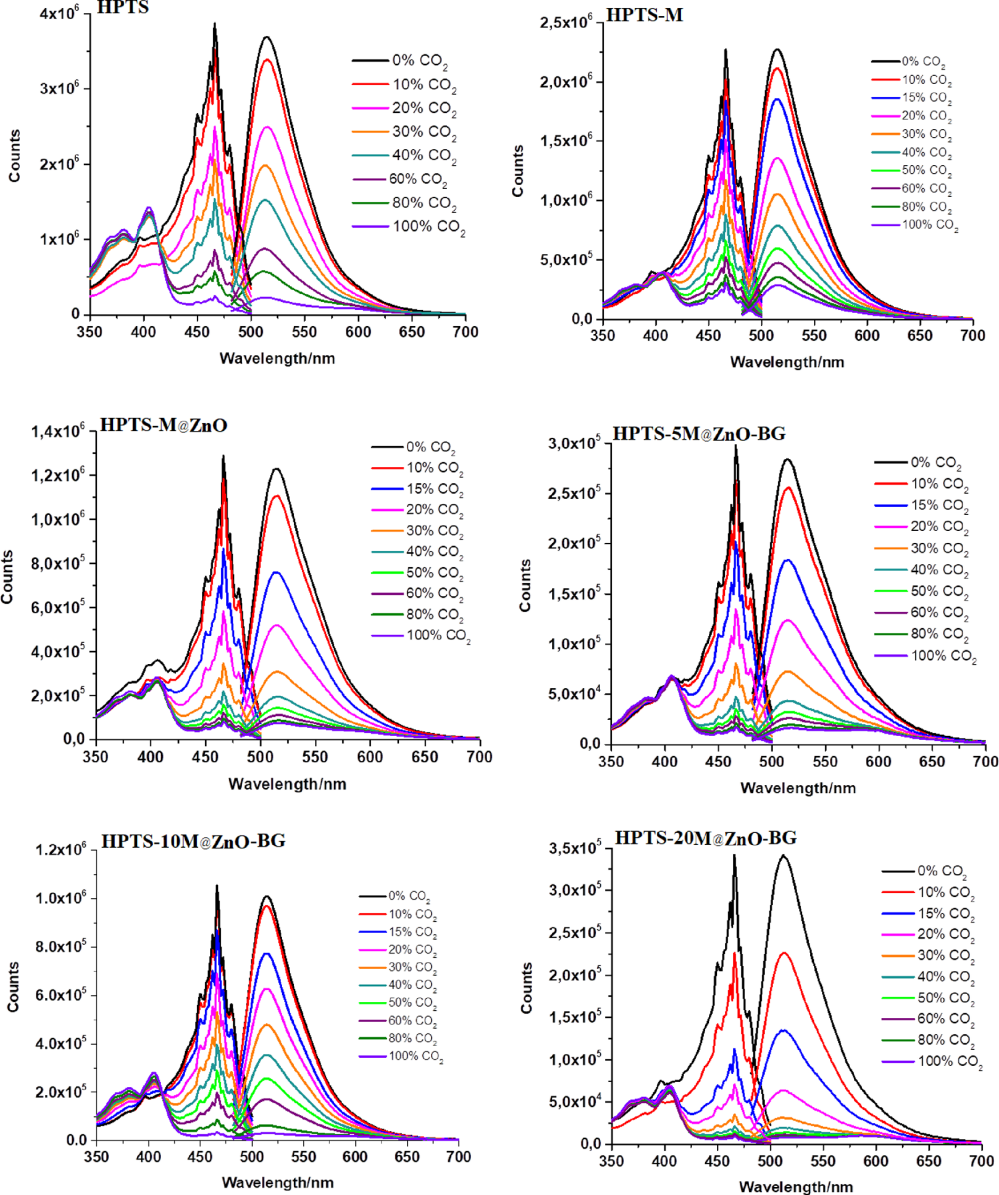
Emission–excitation spectra of the maghemite-based additive-doped HPTS-based composite in PMMA thin films for the 0–100% p[CO_2_] concentration range.

Figure 5 shows the changes in the emission and absorption spectra of HPTS-based composites in the presence of M@ZnO-BG-based additives in the partial pressure of CO_2_ (p[CO_2_]) concentration range of 0–100%.

**Figure 6:**
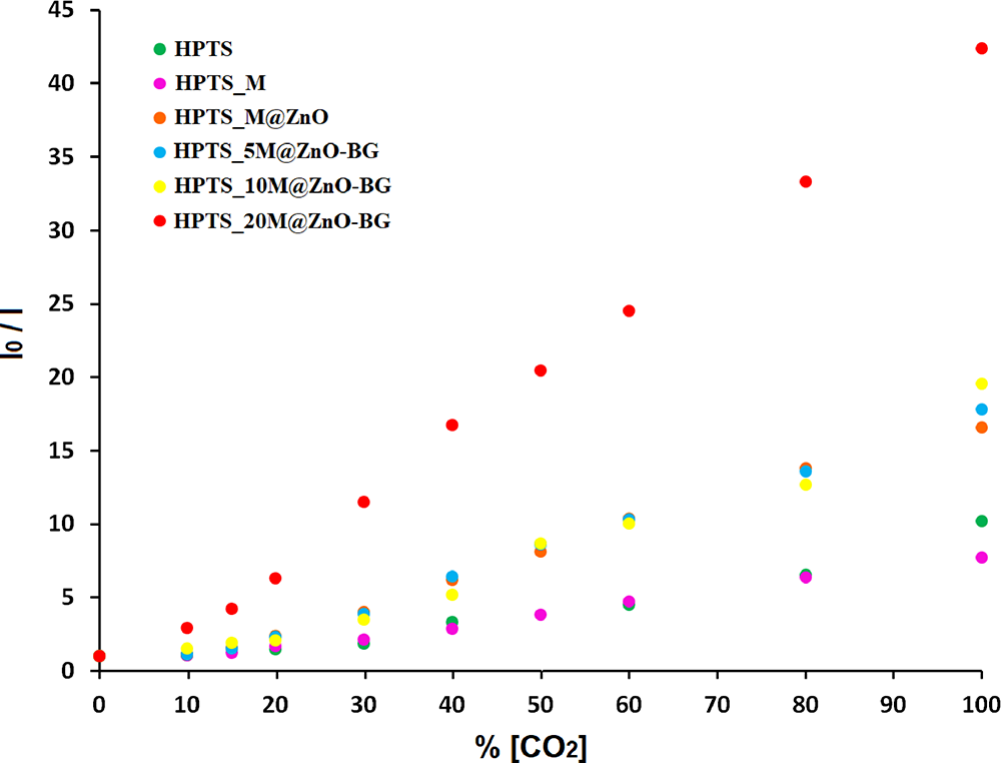
Comparative calibration curves for the 0–100% p[CO_2_] concentration range for all HPTS-based composites.

The calibration graphs of the examined thin films are shown in Figure 6 for a comparison of the responses obtained from various films over the concentration range of 0–100% p[CO_2_]. However, Table 3 indicates the calibration equations, Stern–Volmer constants (*K*_sv_), regression coefficients (*R*^2^), and sensor sensitivity (*I*_0_/*I*_100_) values for the HPTS-based composites in the 0–100% [CO_2_] concentration range. The linear equation and correlation coefficient for the additive free-HPTS thin film were determined as *y* = 0.0741 + 1 and 0.8914 in the specified CO_2_ concentration range, respectively. It appears that the HPTS_20M@ZnO-BG sensor material exhibited a more significant slope and superior linear response compared to those of the other composites when examining the CO_2_-induced variations in the 0–100% p[CO_2_] concentration range.

**Table 3:** Optical Properties and Carbon Dioxide Sensitivity of the HPTS–Based Composites

**sample**	**eq** (0–100**% [CO**_**2**_**])**	**Stern–Volmer constant (*K***_**sv**_**)**	***R***^**2**^	***I***_**0**_**/*I***
HPTS	*y* = 0.0741*x* + 1	7.41 × 10^–2^	0.8914	10.2
HPTS_M	*y* = 0.0622*x* + 1	6.22 × 10^–2^	0.9505	7.7
HPTS_M@ZnO	*y* = 0.1492*x* + 1	14.92 × 10^–2^	0.9590	16.6
HPTS_5M@ZnO-BG	*y* = 0.1544*x* + 1	15.44 × 10^–2^	0.9547	17.8
HPTS_10M@ZnO-BG	*y* = 0.1557*x* + 1	15.57 × 10^–2^	0.9228	19.6
HPTS_20M@ZnO-BG	*y* = 0.3976*x* + 1	39.76 × 10^–2^	0.9864	42.4

In this study, the use of M@ZnO-BG particles with HPTS in the PMMA matrix resulted in improved *I*_0_/*I*_100_ values compared with previous studies in the literature. It was seen that the enhancement of the CO_2_ sensitivity resulted from adding these additives to the dye. While the *I*_0_/*I*_100_ value indicating the sensor sensitivity for HPTS without additives was 10.22, these values were found to be between 7.73 and 42.40 along with M@ZnO-BG additives (see Table 3). It was observed that HPTS_20M@ZnO-BG thin films increased the sensitivity to carbon dioxide gas ∼4 times compared to the additive-free form.

### Interactions between the HPTS and Additives

**Figure 7:**
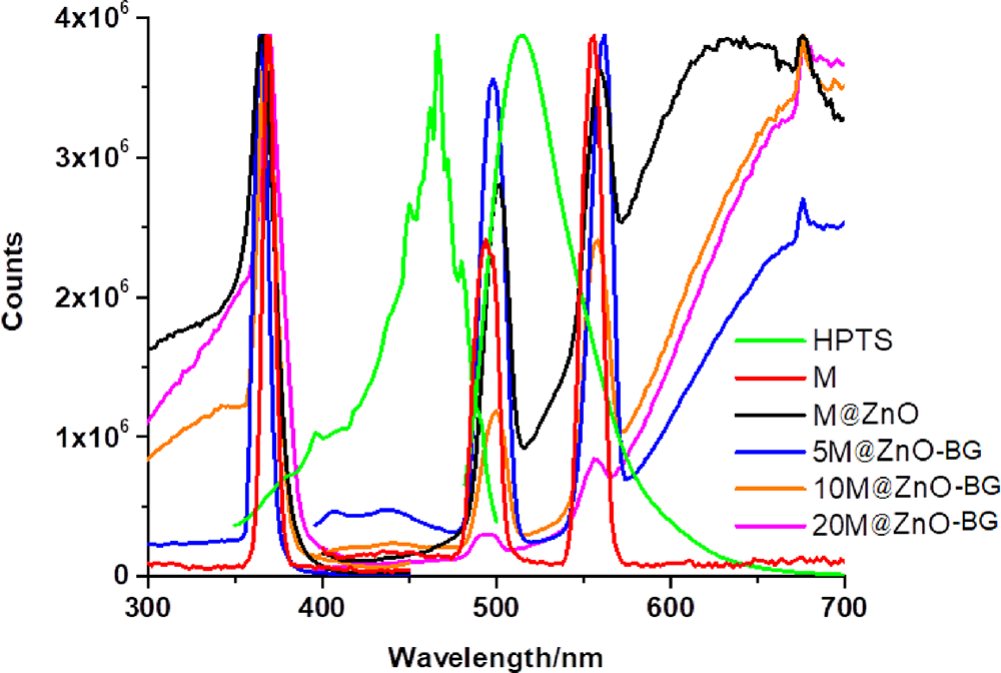
Excitation and emission spectra of the PMMA-embedded HPTS, M, M@ZnO, 5M@ZnO-BG, 10M@ZnO-BG, and 20M@ZnO-BG NPs.

The response of the sensor is greatly influenced by the interaction between the detecting element and the gas being detected. We individually recorded the excitation and emission spectra of the HPTS and maghemite-based NPs to better understand the causes of the related improvement (Figure 7). As it was seen, the M, M@ZnO, and M@ZnO-BG NPs have absorption and release properties within 270 and 450 and 400 and 700 nm wavelength ranges, respectively. These ranges overlap with the excitation band of HPTS, suggesting the possibility of energy transfer between the nanoparticles and HPTS. Energy transfer, also referred to as Förster resonance energy transfer (FRET), is the term used to describe the phenomena. FRET occurs when two fluorophores (light-absorbing molecules) are in close proximity, and the excitation energy from one fluorophore is transferred to the other through nonradiative dipole–dipole interactions. In this case, the nanoparticles and HPTS act as potential energy donors and acceptors, respectively.

### Decay Time Measurements

**Figure 8:**
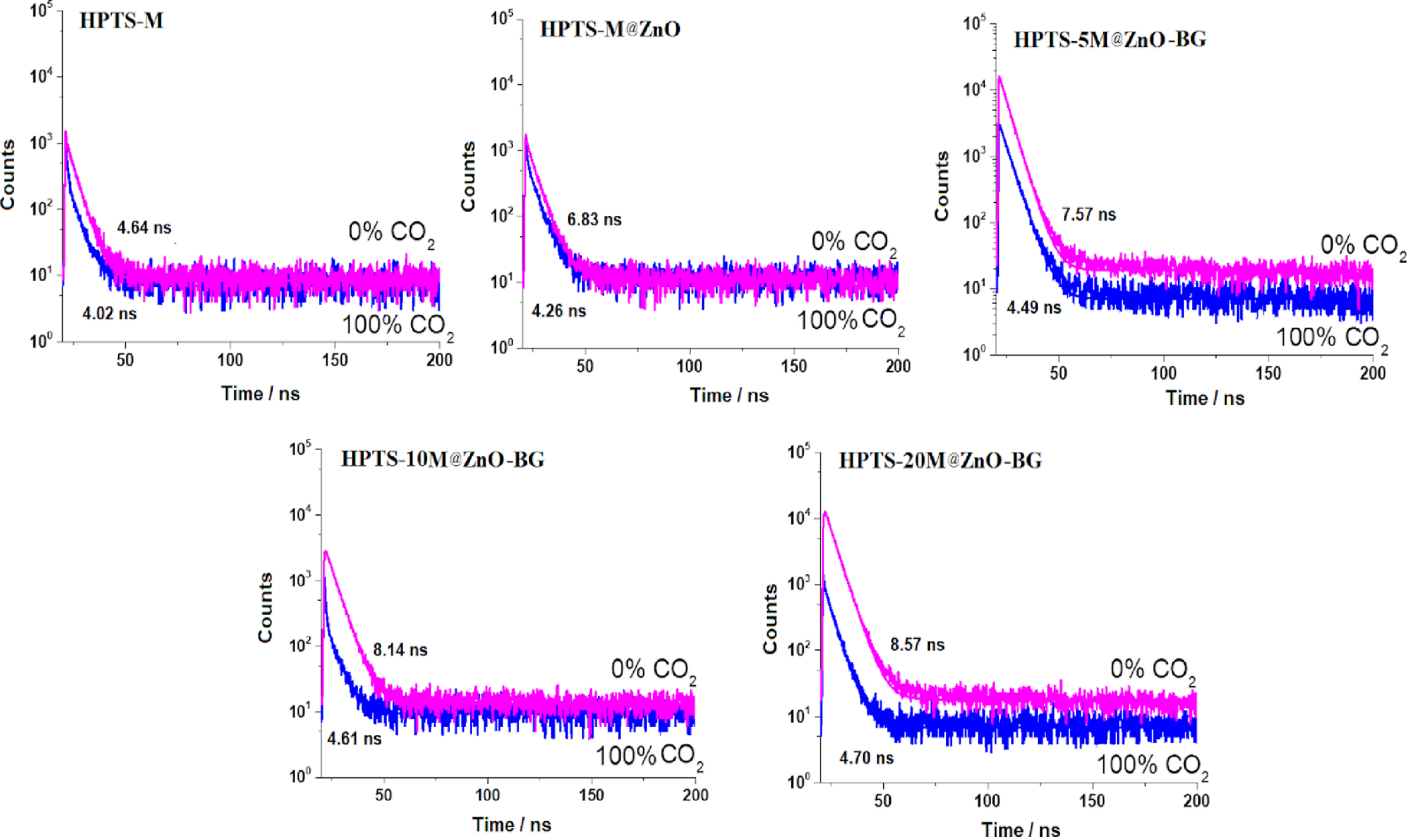
Decay curves of (top left) HPTS_M, (top middle) HPTS_M@ZnO, (top right) HPTS_5M@ZnO, (bottom left) HPTS_10M@ZnO, and (bottom right) HPTS_20M@ZnO in the fully N_2_ and CO_2_ gas atmospheres.

Longer or shorter decay times in the presence of CO_2_ can occur, which affects the fluorescence dynamics like energy transfer, fluorescence quenching, or other interactions. The decay time values were measured for the HPTS dye when combined with M@ZnO-BG. Table 4 shows all of the recorded decay time values. Measuring the decay kinetics of HPTS-based thin films gives us important information about the interaction mechanism between the quencher and fluorophore (Figure 8).

**Table 4:** Decay Time Values of the HPTS-Based Sensing Agents

**sample**	**τ**_**O**_**(0% CO**_**2**_**)**	**decay time (ns)**	**std. dev. (ns)**	**rel. (%)**	**τ**_**O**_**(100% CO**_**2**_**)**	**decay time (ns)**	**std. dev. (ns)**	**rel. (%)**
HPTS_M	τ_1_	0.93	0.03	0.20	τ_1_	0.61	0.03	15.54
	τ_2_	5.53	0.01	0.80	τ_2_	4.64	0.01	84.46
	τ_avr_	4.64			τ_avr_	4.02		
HPTS_M@ZnO	τ_1_	2.28	0.02	21.55	τ_1_	0.60	0.01	13.78
	τ_2_	8.08	0.03	78.45	τ_2_	4.69	0.02	86.22
	τ_avr_	6.83			τ_avr_	4.26		
HPTS_5M@ZnO	τ_1_	2.87	0.01	22.74	τ_1_	1.65	0.02	11.34
	τ_2_	8.95	0.02	77.26	τ_2_	4.86	0.02	88.66
	τ_avr_	7.57			τ_avr_	4.49		
HPTS_10M@ZnO	τ_1_	3.01	0.01	25.58	τ_1_	1.75	0.03	11.62
	τ_2_	9.90	0.03	74.42	τ_2_	4.99	0.02	88.38
	τ_avr_	8.14			τ_avr_	4.61		
HPTS_20M@ZnO	τ_1_	3.85	0.01	22.53	τ_1_	1.95	0.01	10.20
	τ_2_	9.95	0.02	71.47	τ_2_	5.01	0.02	89.80
	τ_avr_	8.57			τ_avr_	4.70		

While the fluorescence decay time values of HPTS_M@ZnO and HPTS_20M@ZnO-BG were recorded as 4.64 and 8.57 ns in the N_2_ atmosphere, all of the phosphorescence decay time values were reduced to 4.02 and 4.70 ns when fully exposed to CO_2_, respectively (see Table 4). When combined with the HPTS_20M@ZnO-BG additive, the multiple exponential decay time values of HPTS exhibited a greater decrease than the other forms. Certain factors, namely, surface defects, electrical conductivity, and charge transfer, have an impact on the decay time kinetics. These factors contribute to a reduction in the rate at which decay occurs. The presence of adsorbed or diffused gas in maghemite-doped ZnO with bioactive glass additives has several consequences, including a decrease in carrier density, potential barriers between oxide particles leading to reduced electrical conductivity, a decrease in luminescence intensity, and slower decay time kinetics. These effects highlight the importance of understanding and controlling the gas environment and its impact on the material’s properties to optimize its performance.

### Reproducibility of the Sensor Slides

**Figure 9:**
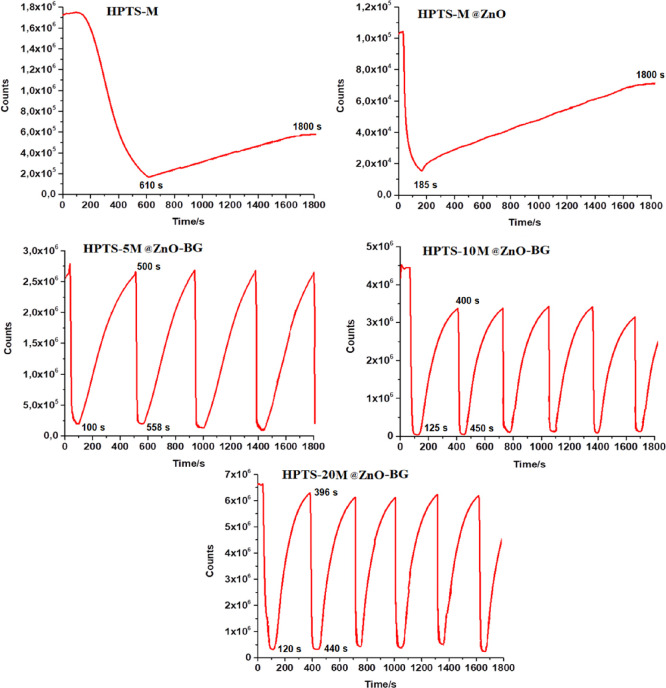
Kinetic response of (top left) HPTS_M, (top right) HPTS_M@ZnO, (middle left) HPTS_5M@ZnO, (middle right) HPTS_10M@ZnO, and (bottom) HPTS_20M@ZnO in the fully N_2_ and CO_2_ gas atmospheres.

The presence of maghemite-doped ZnO and bioactive glasses may alter the surface properties of the sensing material, leading to improved adsorption–desorption kinetics. This can result in faster desorption of the CO_2_ molecules from the surface, thereby reducing the recovery time. The regeneration measurement is a crucial parameter for the application of CO_2_ sensor applications. It assesses the ability of a sensor to recover its sensing properties after exposure to CO_2_. The regeneration process is important because it allows the sensor to be reused for multiple sensing cycles, ensuring its long-term functionality and reliability. The response and reversibility measurements of the PMMA thin film embedded HPTS dye in the presence of M@ZnO-containing bioactive glasses were interpreted according to time and varying quencher concentrations in fully N_2_ and CO_2_ gas atmospheres. The results were obtained as response time and reversibility for CO_2_ sensing (see Figure 9).

In the literature, the 50% response and recovery times of the HPTS implanted in the LDPE thin film on exposure to 5.0% CO_2_ were described as 120 and 2340 s by Mills and Yusufu, respectively.^4^ Oter et al. found the response and the regeneration time of 1-methyl-3-butyl imidazolium tetrafluoroborate and 1-methyl-3-butyl imidazolium bromide as in the ranges of 1–2 and 7–10 min on exposures to 0.0–10 and 0–60% CO_2_, respectively.^13^ In this work, the response and recovery times of HPTS_20M@ZnO-BG were determined as 44 and 276 s in fully CO_2_ and N_2_ atmospheres, respectively. The specific role of M@ZnO-BG additives in achieving reversible behavior is dependent on the composition and interaction dynamics of the materials involved. Among the sensor agents, HPTS_20M@ZnO-BG showed good repeatability and stability during multiple sensing cycles. The signal changes of HPTS_20M@ZnO-BG were reversible during measurements after the fifth cycle. Additionally, the standard deviations of the upper and lower signal intensities were found to be less than 5.0%.

The long-term stability of HPTS-based optical sensors was improved by using an ionic liquid during the preparation of thin films. By introducing an ionic liquid, it is possible to enhance the robustness of the thin film matrix and prevent degradation of the fluorescent dye over time, resulting in improved sensor stability. Ongun reported the long-term stability of HPTS dye-based thin films as 2 months in the presence of ZnO and ZnO@Ag NPs stored under ambient conditions in the laboratory.^12^ Aydogdu et al. reported that there was no significant deviation in signal intensity after 7 months when electrospun nanofibers of HPTS embedded in PMMA and EC matrices were stored in the ambient air of the laboratory.^41^ Oter et al. reported the shelf life of EMIM BF_4_-doped HPTS-based thin films as 95 days.^13^ To evaluate the long-term stability of stock sensor materials prepared with EMIMBF_4_ and EC, Celik and his co-workers tested them with 10^–5^ M NaHCO_3_ solution every day and did not see any signal shift after 195 days.^42^ In this study, the HPTS-based sensing slides kept in the dark in a lab environment for 10 months still displayed approximately the same emission-based intensity. The obtained results led us to the conclusion that all sensing membranes provide consistent and repeatable CO_2_ measurement results.

## Conclusions

In the study, γ-Fe_2_O_3_ nanoparticles were synthesized using the coprecipitation method, and the γ-Fe_2_O_3_@ZnO core–shell nanostructures were fabricated. Silicate-based 13–93 bioactive glass powders containing the γ-Fe_2_O_3_@ZnO magnetic phase at different concentrations were prepared through the sol–gel process. Results revealed that the ZnO coating layer improved the dispersion ability of the agglomerated maghemite nanoparticles. Overall, the addition of bioactive glass particles to the γ-Fe_2_O_3_@ZnO material brought about structural improvements such as increased porosity, enhanced stability, controlled release properties, and surface functionalization. As a result, the pH-sensitive HPTS dye was employed here for the first time with γ-Fe_2_O_3_/ZnO-containing BG additions, and it was discovered that doing so increased the dye’s sensitivity to CO_2_ gas. The *I*_0_/*I*_100_ value of free-HPTS was 10.2, but it grew 4-fold when the 20M@ZnO-BG additive was present. Furthermore, HPTS-20M@ZnO-BG has a superior linear calibration graph, larger *K*_sv_ constant, better relative signal change, and higher sensitivity to CO_2_ gas in the 0–100% p[CO_2_] range. These enhancements can result in improved performance and expanded application potential for the γ-Fe_2_O_3_/ZnO-containing BG particle-doped HPTS composite materials. They can be used as a promising sensor agent in the detection of CO_2_ gas in areas such as catalysis, gas-sensing, biomedical engineering, and environmental applications.

## Data Availability

Data will be made available on request.
